# Periodontal Phenotype Modification Using Subepithelial Connective Tissue Graft and Bone Graft in the Mandibular Anterior Teeth with Mucogingival Problems Following Orthodontic Treatment

**DOI:** 10.3390/medicina59030584

**Published:** 2023-03-16

**Authors:** Won-Bae Park, Wonhee Park, Seung-Weon Lim, Ji-Young Han

**Affiliations:** 1 Department of Periodontology, School of Dentistry, Kyung Hee University, Seoul 02447, Republic of Korea; 2 Private Practice in Periodontics and Implant Dentistry, Seoul 02771, Republic of Korea; 3 Department of Dentistry, Division of Dentistry, College of Medicine, Hanyang University, 222-1 Wangsimni-ro, Seongdong-gu, Seoul 04763, Republic of Korea; 4 Department of Orthodontics, Division of Dentistry, College of Medicine, Hanyang University, 222-1 Wangsimni-ro, Seongdong-gu, Seoul 04763, Republic of Korea; swlim@hanyang.ac.kr; 5 Department of Periodontology, Division of Dentistry, College of Medicine, Hanyang University, 222-1 Wangsimni-ro, Seongdong-gu, Seoul 04763, Republic of Korea

**Keywords:** bone graft, gingival recession, orthodontic treatment, subepithelial connective tissue graft

## Abstract

Among the complications of orthodontic treatment, mucogingival problems with gingival recession in the mandibular anterior teeth are challenging for clinicians. Mucogingival problems can lead to esthetic deficits, thermal hypersensitivity, tooth brushing pain, and complicated plaque control. Herein, we present a case of a 16-year-old female with gingival recession in the left mandibular central incisor after orthodontic treatment. The preoperative clinical findings showed a thin soft tissue biotype with root prominence in the mandibular anterior area. The interdental area was relatively depressed. After reflection of the full-thickness flap, root coverage using a bone graft substitute and subepithelial connective tissue graft obtained from the palatal mucosa was performed. The 6-month and 5-year postoperative clinical findings showed improved soft tissue phenotype. The cross-sectional CBCT scans 5 years after surgery showed a well-maintained labial bone plate in the mandibular incisors. Within the limitations of this case report, for patients with gingival recession in the mandibular incisors after orthodontic treatment, a successful biotype modification can be achieved with a combined procedure using subepithelial connective tissue graft with bone graft substitutes.

## 1. Introduction

Gingival recession is associated with attachment loss and exposure of the root surface to the oral environment [1]. Gingival recession causes impaired esthetics, dentin hypersensitivity, root caries, non-carious cervical lesions, and complicated plaque control [1,2]. The causes of gingival recession vary. A thin periodontal biotype, improper tooth brushing, frenal attachment, absence of attached gingiva, and reduced thickness of the alveolar bone due to abnormal tooth position are predisposing factors for gingival recession [1,3,4]. In addition, orthodontic treatment can cause gingival recession depending on the direction of orthodontic force [1,3,5,6]. When orthodontic forces move teeth out of the alveolar bone housing, particularly in the labial direction, alveolar bone dehiscence, reduced buccolingual tissue thickness, and gingival recession can occur [4,5,7,8,9]. Therefore, orthodontists should strive to prevent mucogingival problems during or after orthodontic treatment [10].

Various techniques including a free gingival graft, a subepithelial connective tissue graft, and a coronally advanced flap have been introduced for increasing the width of keratinized mucosa or covering the denuded root surface [11,12]. In root coverage procedures for patients with gingival recession, a coronally advanced flap with a subepithelial connective tissue graft is considered the gold standard [11,13]. In addition, other procedures including guided tissue regeneration [14], tunnel technique [15,16], and partly epithelized connective tissue graft [17,18] have been reported. As an alternative to autogenous soft tissue graft, acellular dermal matrix, and collagen matrix are also used [19].

Although various techniques have been used for the treatment of gingival recession after orthodontic treatment [16,20], to the best of the authors’ knowledge, the use of a bone graft substitute with a subepithelial connective tissue graft for treatment of gingival recession in the mandibular incisors has not been reported. Herein, a patient case with a complex mucogingival problem accompanied by thin periodontal biotype and labial root prominence in which the gingival recession was treated with a bone graft substitute and a subepithelial connective tissue graft is reported.

## 2. Case Report

A 16-year-old female sought esthetic improvement of the mandibular anterior area. She was referred to our clinic to treat gingival recession of the lower anterior teeth. Clinically, a severe gingival recession on the lower left central incisor was observed. The patient had no history of systemic diseases. She had an orthodontic treatment to resolve mandibular anterior crowding. She had undergone orthodontic treatment for about 2 years.

**Figure 1:**
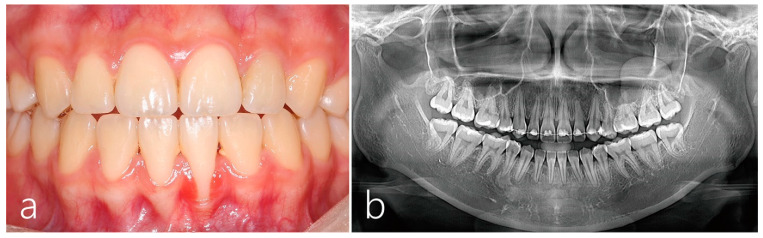
(**a**) Clinical finding 2 years after orthodontic treatment showed gingival recession in the left lower central incisor with a thin periodontal biotype. The roots of lower incisors were slightly labially protruded, and the interdental areas were relatively depressed. (**b**) Root resorption and interdental bone loss of lower incisors were observed on panoramic radiography at 2 years after orthodontic treatment.

The preoperative clinical findings showed a thin soft tissue biotype with root prominence in the mandibular anterior area. The interdental area was relatively depressed (Figure 1a). Gingival recession of the lower left central incisor extended to the mucogingival junction. The mobility was evaluated using Periotest Classic (Medizintechnik Gulden e. K., Modautal, Germany). The Periotest value (PTV) of the lower left central incisor was 9. The patient complained of tooth hypersensitivity and discomfort in the lower left central incisor during tooth brushing. During initial treatment, an intraoral tooth brushing instruction was performed for the improvement of oral hygiene. The patient was asked to bring her toothbrush for oral hygiene instruction. However, the patient’s oral hygiene was poor, although initial treatment including scaling was performed, and repeated oral hygiene instructions were provided. Preoperative panoramic radiography showed slight root resorption of mandibular incisors. In addition, loss of interdental bone between the lower right and left central incisors were observed on preoperative panoramic radiography (Figure 1b). Informed consent form was received from the patient before surgery.

**Figure 2:**
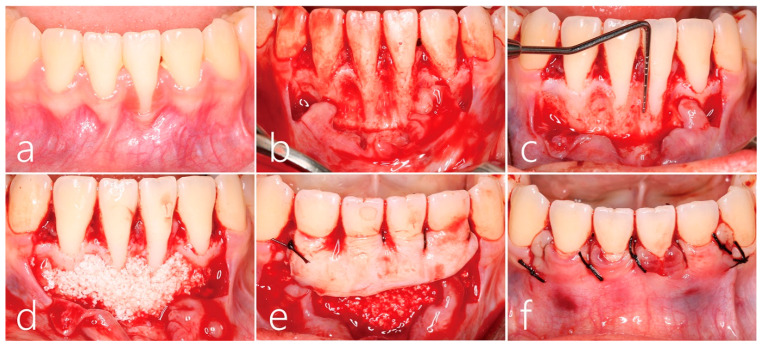
(**a**) Preoperative clinical finding showed gingival recession of the left lower central incisor extended to the mucogingival junction. (**b**) After reflection of a full-thickness flap on the labial side, root planing was performed. (**c**) The labial dehiscence of the left lower central incisor was >7 mm from the cemento-enamel junction, and interproximal concavity was observed. (**d**) The interproximal concavity and thin labial bony plate were filled with a synthetic bone graft substitute. (**e**) The subepithelial connective tissue graft obtained from the left palate using the trap-door approach was fixed with a 5-0 catgut suture slightly above the cemento-enamel junction. (**f**) The overlying flap was closed with 5-0 black silk to ensure the subepithelial connective tissue graft was covered.

Signs of slight inflammation with supragingival plaque and calculus were observed before surgery (Figure 2a). Under local anesthesia with lidocaine containing 1:100,000 epinephrine, vertical incisions at the mesial line angle of the left and right canines were made beyond the mucogingival junction while preserving the lingual interdental papilla. The full-thickness flap was carefully reflected to minimize the trauma using a periosteal elevator (Allen Periosteal Elevator, Anterior, Hu-Friedy Mfg. Co., Chicago, IL, USA). Then, the bone graft substitute was added to the inter-root concavity (Figure 2b). The labial dehiscence of the lower left central incisor was >7 mm from the cemento-enamel junction (Figure 2c). After root planing, the interproximal concavity and thin labial bony plate were filled with synthetic bone graft substitute (Osteon III, Genoss, Suwon, Republic of Korea; Figure 2d). A 1.5 mm thickness of subepithelial connective tissue graft harvested from the left palate using the trap-door approach was fixed with a 5-0 catgut suture slightly above the cemento-enamel junction (Figure 2e). The overlying flap was closed with 5-0 black silk to ensure the subepithelial connective tissue graft was covered (Figure 2f). All sutures were removed after 10 days. Healing of both donor and recipient sites was uneventful.

**Figure 3:**
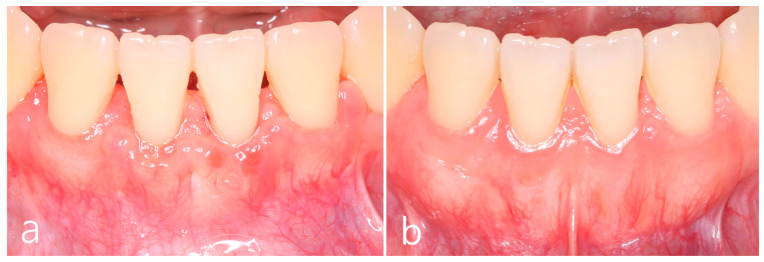
(**a**) Clinical findings at 6 months after surgery showed reduced gingival recession in the left lower central incisor and improved soft tissue phenotype. (**b**) The 5-year postoperative clinical finding showed improved soft tissue phenotype. Interdental concavity was also improved.

The clinical outcomes 6 months after surgery showed reduced gingival recession of the lower left central incisor. In addition, soft tissue phenotype was improved. Color discrepancy was not observed, but some interdental space with supragingival plaque and calculus was observed (Figure 3a). The 5-year postoperative clinical finding showed improved soft tissue phenotype. However, the patient’s oral hygiene was not improved (Figure 3b).

**Figure 4:**
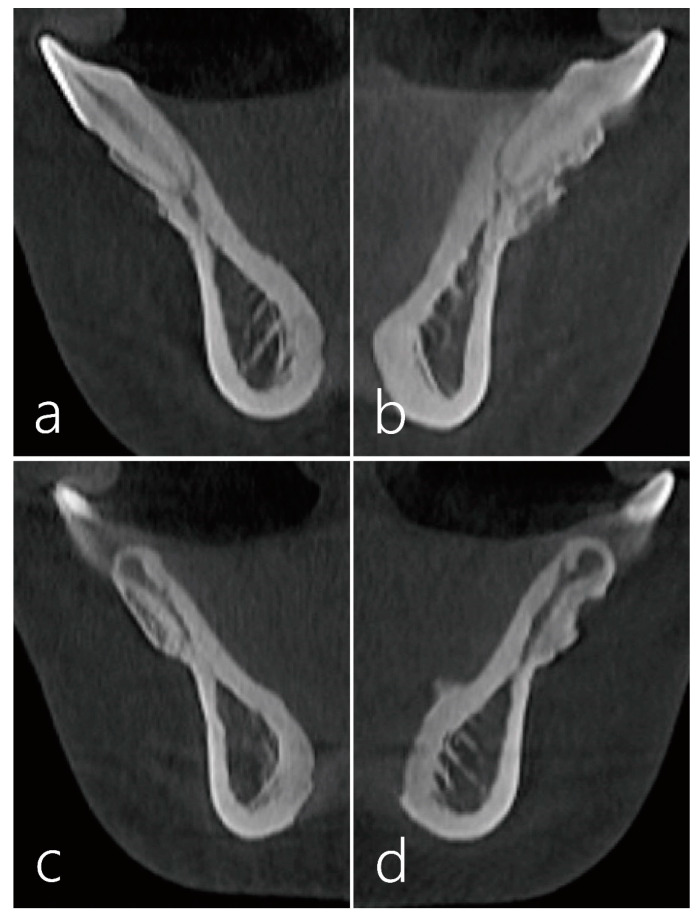
(**a**,**b**) The cross-sectional CBCT scans 5 years after the surgery showed well maintained labial bone plate at the midfacial side of both mandibular central incisors. (**c**) On the cross-sectional CBCT scan at the interdental site between the right lower central and lateral incisors, hard tissue phenotype modification was observed on the labial side. (**d**) On the cross-sectional CBCT scan at the interdental site between the left lower central and lateral incisors, partial depression was present but hard tissue phenotype was improved overall.

The cross-sectional CBCT scans 5 years after the surgery showed well maintained labial bone plate at the midfacial side of the lower right and left central incisors (Figure 4a,b). On the cross-sectional CBCT scan at the interdental site between the lower left central and lateral incisors, partial depression was present, although the hard tissue phenotype improved overall (Figure 4c). On a cross-sectional CBCT scan, hard tissue phenotype modification was observed on the labial side at the interdental site between the lower right central and lateral incisors (Figure 4d).

## 3. Discussion

The present case showed a successful clinical outcome of a subepithelial connective tissue graft and bone graft procedure performed on the lower incisors with thin periodontal biotype and gingival recession after orthodontic treatment. In addition, the cross-sectional CBCT scans showed that the interdental concavity was augmented with bone graft substitutes combined with a subepithelial connective tissue graft.

Gingival recessions in the lower anterior teeth after orthodontic treatment are challenging for periodontists. Gingival recession may compromise outcomes of orthodontic treatment and adversely affect dentofacial esthetics or cause tooth hypersensitivity [21]. Animal studies showed that labial movement of the lower incisors in monkeys caused bone dehiscence and subsequent loss of periodontal attachment [22,23]. Conversely, in a clinical study, gingival recession in the lower incisors was reduced with orthodontic correction of the root toward the center of the alveolar envelope [24]. The direction of tooth movement and the thickness of gingiva may play important roles in soft tissue alteration during and after orthodontic treatment [1,5,25]. Reportedly, gingival augmentation is needed in areas with <2 mm of keratinized gingiva before orthodontic treatment [1,25,26]. In this case, the lower incisors slightly contact with the upper incisors when the mandible is protruded. A patient’s occlusal relationship should be considered because the orthodontic force applied to the root outward from the center of the alveolar envelope may increase gingival recessions [22,24].

In addition, Wennström emphasized the importance of proper plaque control before, during, and after orthodontic treatment [5]. In the present case, the patient had supragingival plaque and calculus on the lower incisors immediately before surgery despite repeated oral hygiene instructions. The gingival recession with a thin periodontal biotype extending to the cemento-enamel junction may affect the patient’s poor oral hygiene in this case.

In several root coverage procedures, a coronally advanced flap with a subepithelial connective tissue graft is considered the gold standard [11,13]. In addition, alternatives such as acellular dermal matrix and collagen matrix are used with a coronally advanced flap to reduce morbidity at the donor site [27,28,29]. Although some clinical advantages have been reported, this procedure is not as effective as a coronally advanced flap with a subepithelial connective tissue graft [28,30]. Therefore, a subepithelial connective tissue graft was used in this patient. In addition, all lower incisors had a thin periodontal phenotype with interproximal bony concavity. Therefore, we extended the flap to the mesial line angles of both canines instead of a localized flap. A wide subepithelial connective tissue graft was acquired using the trap-door approach. Although healing was prolonged due to the large donor site, the patient’s satisfaction with the clinical outcome was high.

We did not perform a root conditioning procedure before using a subepithelial connective tissue graft with bone substitute. Various root modifiers including different root conditioners, lasers, EMD, recombinant human growth factors, and platelet-rich plasma have been used to improve the healing process and increase the success rate of root coverage [31]. There is controversy in using root modifiers for root coverage procedures. Several authors suggested that this procedure requires a considerable amount of time and costs and justification of its use should be considered [31,32]. The relative influence of mechanical or chemical treatment of the root surface for complete root coverage has been questioned [32]. A systematic review concluded that EDTA may be beneficial in improving the clinical outcomes of root coverage using a coronally positioned flap with subepithelial connective tissue graft [33]. However, it also is suggested that the time and cost of using EDTA must be considered [33]. In addition, a recent clinical study showed that root conditioning using EDTA did not improve outcomes of root coverage with a subepithelial connective tissue graft [34]. 

In addition, bone graft substitutes were used to reduce interproximal concavity. For dehiscence-type defects, an animal study showed that biphasic hydroxyapatite + beta tricalcium phosphate (ß-TCP) or deproteinized bovine bone mineral may provide an osteoconductive scaffold to support guided bone regeneration procedures [35]. In addition, histologic findings of the combination of hydroxyapatite and ß-TCP showed the newly formed primary spongy woven bone invaded the defect area bone [35,36]. We used synthetic bone substitute because of its unique property. The ß-TCP matrix contains biocompatible bone-like tissue components with a good balance between degradation and resorption during bone formation [37]. As the denuded root surface is avascular, we were concerned that the bone graft particles on the denuded root surface may protrude through the gingiva and be perceived as a foreign body. However, the cross-sectional CBCT scans showed well-consolidated bone-like tissue on the root surface and interproximal concavity. Consequently, interdental depression was also resolved, resulting in reduced root prominence of the mandibular incisors. In the present case, a subepithelial connective tissue graft may have acted as a barrier membrane. The use of a subepithelial connective tissue graft as a barrier membrane has been reported in several studies [38,39,40]. However, future well-controlled clinical studies with a large sample size are needed to evaluate the clinical efficacy of a subepithelial connective tissue graft with bone graft substitutes for root coverage of gingival recession in the mandibular incisors.

## 4. Conclusions

Within the limitations of the present case report, for patients with thin soft and hard tissue phenotype of the mandibular anterior region and gingival recession that occur due to complications of orthodontic treatment, a successful periodontal phenotype modification can be achieved with a combined procedure using a subepithelial connective tissue graft with bone graft substitutes. In addition, interdental concavities between the lower incisors were also improved.

## Data Availability

Not applicable.
